# Transoral thyroidectomy-learning curve

**DOI:** 10.20945/2359-3997000000382

**Published:** 2021-06-29

**Affiliations:** 1 Memorial Sloan Kettering Cancer Center New York Department of Surgery New York USA Head and Neck Service, Department of Surgery, Memorial Sloan Kettering Cancer Center New York, New York.

Professor Bertelli and cols. from Brazil have reported their early experience and complications during learning curve of transoral endoscopic thyroidectomy vestibular approach (TOETVA) (1). The authors have described their early experience of multiple institutions in Brasil having learned TOETVA either in Korea or in John Hopkins-Baltimore. They developed a study group, TOETVA-Bra. Two of the authors are from Johns Hopkins who have larger experience. The authors have reported a collective early experience of 93 patients undergoing TOETVA. Fifty-eight percent had total thyroidectomy, while 59% had benign pathology. The authors have reported 21.5% complications, 16% of which were minor.

It is commendable to organize a study group and report early experience with specific focus on safety and outcomes of adopting a new surgical procedure. The authors have not given the denominator from which these 93 patients were derived. However, at least 7 operating surgeons were directly involved in these procedures. They have used the same technique as popularized by Anuwong from Thailand (2). Even though the authors have titled the manuscript as “Learning Curve” it remains unclear as to the number of procedures required during the learning curve. The complication rate described by the authors is similar to many other publications on this subject. This technique was initially popularized in Thailand and subsequently commonly adopted in Korea and a few centers in the United States. However, there seems to be considerable interest in this surgical approach. The authors have used this for both benign and malignant problems. Approximately 2,000 transoral thyroidectomies have been performed worldwide, of which 400 were in the United States (3).

The entire idea of endoscopic thyroidectomy is to avoid a cervical scar. Whether avoiding a neck incision should be considered as a major indication for endoscopic thyroidectomy remains unclear. Initially the scarless thyroidectomy started in Korea, with transaxiliary approach and robotic instrumentation. There was a huge experience in Korea and selected centers in United States. The transaxiliary robotic surgery did not become very popular in United States partly due to higher rate of complications and a high learning curve and non approval of robotic instrumentation for thyroidectomy by FDA. It appears that there is considerable interest in transoral thyroidectomy; however, what is the exact learning curve remains unclear. And also, how many procedures should a surgeon initially perform under direct supervision of an expert mentor. The indications for this approach remain somewhat unclear and there is always a concern about lip issues and mental nerve injury. It is interesting to note that there are 324 publications on this subject in PubMed, most of which are in the last five years.

It may be difficult to undertake these procedures without true hands-on training in a specialized center where these procedures are commonly performed. There are two interesting publications on learning curve for TOETVA. Liang and cols. reported 30 cases as learning curve while Cao and cols. reported 25 cases for learning curve (4,5). Lira and cols. reported a drop in surgical time from 167 minutes to 117 minutes after 15 cases (6). It is interesting to note that the transaxiliary endoscopic surgery had a learning curve of 60 cases (7).

The major question remains where should the surgeons experience this learning curve, in their own institution with start-up of this new procedure or a fellowship hands-on training in a specialized center? We always talk about learning curve in surgery, but sometimes we do not recognize higher incidence of complications during the early learning curve. This is primarily the reason why fellowship training is important for subspeciality expertise and perfecting surgical technique. There is no room for learning curve for a pilot in the airline industry, should we not adopt the same philosophy in human surgical procedures? We never ask the pilot if they are on the learning curve and also the airlines would not permit the learning curve. The entire idea of endoscopic thyroidectomies is truly not minimally invasive but probably maximally invasive and some of the complications may be difficult for the patient and the surgeon to handle. Endoscopic thyroidectomy is an evolving field and its true application outside of the major centers interested in this procedure remains unclear. The specialized instrumentation and robotics are not available in most parts of the world and direct cervical exploration is still the most chosen surgical procedure. However, the technology is having a direct impact in surgery and future will be evaluated as time goes by and surgical expertise developed in these new techniques around the world. The incidence of complications and cost are important considerations in application of the new technology. I would like to congratulate the authors of this manuscript for collaborative study and honest publication of their early experience.
